# Zinc Enhances Cadmium Accumulation in Shoots of Hyperaccumulator *Solanum nigrum* by Improving ATP-Dependent Transport and Alleviating Toxicity

**DOI:** 10.3390/plants13172528

**Published:** 2024-09-09

**Authors:** Jia Zheng, Yukang Yue, Yuting Zhu, Yufeng Wang, Wenwen Zheng, Linfeng Hu, Dianyun Hou, Fayuan Wang, Liming Yang, Hongxiao Zhang

**Affiliations:** 1 College of Agriculture, Henan University of Science and Technology, Luoyang 471023, China; 2 College of Biotechnology, Tianjin University of Science and Technology, Tianjin 300222, China; 3 College of Environment and Safety Engineering, Qingdao University of Science and Technology, Qingdao 266042, China; 4 State Key Laboratory of Tree Genetics and Breeding, Co-Innovation Center for Sustainable Forestry in Southern China, Nanjing Forestry University, Nanjing 210037, China

**Keywords:** cadmium, proteome, *Solanum nigrum*, zinc, transcriptome, transport

## Abstract

*Solanum nigrum* is a cadmium (Cd) and zinc (Zn) accumulator with potential for phytoextraction of soil contaminated with heavy metals. However, how Zn affects Cd accumulation in *S. nigrum* remains unclear. In this study, *S. nigrum* seedlings were treated with 100 μmol·L^−1^ Zn (Zn100), 100 μmol·L^−1^ Cd (Cd100), and the Zn and Cd combination (Zn100+Cd100) for 10 days under hydroponic culture. Compared with Cd100, the Cd content in stems, leaves, and xylem saps was 1.8, 1.6, and 1.3 times more than that in Zn100+Cd100, respectively. In addition, the production of reactive oxygen species in leaves was significantly upregulated in Cd100 compared with the control, and it was downregulated in Zn100. Comparative analyses of transcriptomes and proteomes were conducted with *S. nigrum* leaves. Differentially expressed genes (DEGs) were involved in Cd uptake, transport, and sequestration, and the upregulation of some transporter genes of Zn transporters (*ZIPs*), a natural resistance associated macrophage protein (*Nramp1*), a metal–nicotianamine transporter (*YSL2*), ATP-binding cassette transporters (*ABCs*), oligopeptide transporters (*OPTs*), and metallothionein (*MTs*) and glutathione S-transferase (*GSTs*) genes was higher in Zn100+Cd100 than in Cd100. In addition, differentially expressed proteins (DEPs) involved in electron transport chain, ATP, and chlorophyll biosynthesis, such as malate dehydrogenases (MDHs), ATPases, and chlorophyll *a*/*b* binding proteins, were mostly upregulated in Zn100. The results indicate that Zn supplement increases Cd accumulation and tolerance in *S. nigrum* by upregulating ATP-dependent Cd transport and sequestration pathways.

## 1. Introduction

Cadmium (Cd) can have toxic effects on plants even at trace levels, such as increased oxidative stress, decreased growth and photosynthesis, and reduced uptake of essential elements [1]. Cd is readily absorbed, transported, and accumulated in plant tissues, thereby posing high potential risks to human and animal health [1,2]. Because the chemical properties of Cd ions are similar to those of some trace metal ions, such zinc (Zn) manganese (Mn), Cd is absorbed into root cells via the same membrane transporters as those used for Zn or Mn [3]. Zn is an essential element in plants, but it is also toxic to cells at excess concentrations [4,5]. The association of Cd and Zn in the environment and their chemical similarity can lead to interactions in plants [4,5,6]. For example, Zn alleviates Cd toxicity in rice by modulating photosynthesis, reactive oxygen species (ROS) homeostasis, and differential gene expression [7,8].

Plants evolved detoxification mechanisms based on Cd complexation with low-molecular-weight ligands, including glutathione (GSH), phytochelatins (PCs), and metallothioneins (MTs) [9,10]. GSH, PCs, and MTs are mainly cysteine-rich ligands involved in Cd detoxification in plant cells [11]. PCs are synthesized enzymatically from GSH by PC synthase, and Cd strongly induces the biosynthesis [10]. In plants, Cd-PCs and Cd-GSH complexes are sequestrated in vacuoles by ATP-binding cassette transporter family C members (ABCCs), and they also may be transported to xylem or phloem by ABC family G members (ABCGs) [12]. In contrast to PCs, MTs are gene products that have cysteine-rich domains and bind metal ions such as copper (Cu), Zn, and Cd to protect cells against toxic effects [9,11].

Many Cd hyperaccumulators are also Zn hyperaccumulators, such as *Arabidopsis helleri*, *Noccaea caerulescens*, *Arabis paniculata*, and *Sedum alfredii* [4,13,14,15]. Those plants complex metals with a range of ligands and then compartmentalize them into inactive cellular sites, with organic acids particularly important in Cd and Zn distribution in plants [4,8,10]. Zn is mainly complexed to malate in aerial parts of *A. helleri* [15], and Zn–malate is also accumulated in epidermal cells and trichomes of leaves of *N. caerulescens* [14]. In the hyperaccumulator *S. alfredii*, Zn was accumulated to 2.9% in shoots in a xylem Zn–citrate form under Zn treatment [15]. The tolerance of *A. paniculata* to excess Zn is mainly manifested in increasing energy metabolism and correcting misfolded proteins [16]. Tolerance and accumulation mechanisms of hyperaccumulators can provide promising ideas relevant for phytoremediation of contaminated soils [17].

*Solanum nigrum* is a Cd hyperaccumulator [18,19,20,21] that is an annual weed of the family Solanaceae found worldwide. Compared with other Cd hyperaccumulators [5,13,14], the growth rate of *S. nigrum* is faster, and biomass is higher, indicating great promise for use in phytoremediation [20,22]. *S. nigrum* is extensively studied owing to its excellent metal uptake ability and high tolerance [20,21,23,24]. Metal transporters can have pivotal roles in Cd uptake and accumulation in *S. nigrum*, according to transcriptome analyses [18,25]. Overexpression of *S. nigrum SnYSL3* in *A. thaliana* increases root-to-shoot translocation ratios of Fe and Mn [26], and in *S. nigrum*, Cd is mainly bound to thiol ligands, with Cd complexed with GSH or PCs sequestered in vacuoles [24,27]. In addition, Cd is also partly complexed by organic acids, and the cell wall biosynthesis pathway has a critical role in Cd detoxification in *S. nigrum* [18]. Ammonium can increase *S. nigrum* Cd detoxification ability by reducing Cd influx in the cell walls of roots [19]. In recent years, some studies have found some mechanisms of Cd transport and accumulation in *S. nigrum* [28,29,30]; however, few are related to the effects and molecular mechanism of exogenous Zn on Cd transport and tolerance in *S. nigrum*.

The objectives of this study were (a) to detect the effects of Zn supplementation on Cd accumulation and tolerance in shoots of *S. nigrum*; (b) to identify significantly induced genes or proteins in leaves of *S. nigrum* under Zn, Cd, and the combination; and (c) to reveal the mechanisms underlying Cd transport and accumulation in shoots of *S. nigrum*, as influenced by Zn supplement.

## 2. Results

### 2.1. Zn and Cd Transport and Accumulation in Shoots of S. nigrum

**Figure 1:**
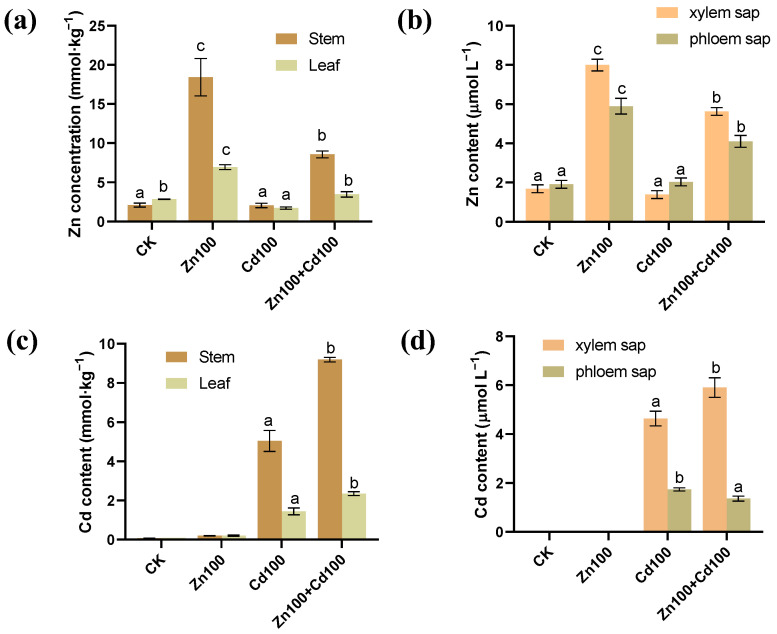
Zn (**a**,**b**) and Cd (**c**,**d**) content in stems, leaves, xylem, and phloem saps of *S. nigrum*. Plants were exposed to a complete Hoagland solution (CK) or with 100 μmol·L^−1^ Zn (Zn100), 100 μmol·L^−1^ Cd (Cd100) and 100 μmol·L^−1^ Zn+100 μmol·L^−1^ Cd (Zn100+Cd100) for 10 days. Values are means ± SE (*n* = 3) of three different experiments. Means denoted by different letters refer to the significant differences (*p* < 0.05, Duncan’s test).

The highest Zn concentrations in stems, leaves, and xylem and phloem saps of *S. nigrum* were found for the 100 μmol·L^−1^ Zn treatment (Zn100), followed by those in the 100 μmol·L^−1^ Zn and 100 μmol·L^−1^ Cd compound treatment (Zn100+Cd100) (Figure 1a,b). In CK and Zn100, almost no Cd was detected, but in the 100 μmol·L^−1^ Cd treatment (Cd100), Cd accumulation was 5.04 mmol·kg^−1^ in stems, 1.43 mmol·kg^−1^ in leaves, 4.64 μmol·L^−1^ in xylem saps, and 1.74 μmol·L^−1^ in phloem saps. In Zn100+Cd100, Cd concentrations in stems, leaves, and xylem saps were 1.8, 1.6, and 1.3 times more than those in Cd100, respectively (Figure 1c,d).

### 2.2. Effects of Zn and Cd on ROS Production in Leaves of S. nigrum

**Figure 2:**
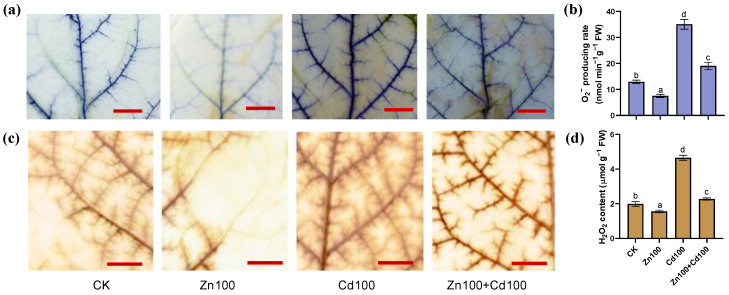
Production of O_2_^−^ (**a**,**b**) and H_2_O_2_ (**c**,**d**) in leaves of *S. nigrum* under Zn and Cd treatment. Histochemical location of O_2_^−^ by NBT staining (**a**) and H_2_O_2_ by DAB staining (**c**), with bar = 1 cm; O_2_^−^ producing rate (**b**) and H_2_O_2_ content (**d**) in leaves of *S. nigrum*. Samples from the second youngest leaf of plants, which were exposed to a complete Hoagland solution (CK) or with 100 μmol·L^−1^ Zn (Zn100), 100 μmol·L^−1^ Cd (Cd100), and 100 μmol·L^−1^ Zn+100 μmol·L^−1^ Cd (Zn100+Cd100) for 10 days. Staining experiments were repeated at least three times, with similar results. Values are means ± SE (*n* = 3) of three different experiments. Means denoted by different letters refer to the significant differences (*p* < 0.05, Duncan’s test).

According to both the histochemical staining and concentration analysis (Figure 2a,d), the production of O_2_^−^ and H_2_O_2_ in leaves of *S. nigrum* was upregulated in Cd100 but downregulated in Zn100, whereas there was a neutralization effect in Zn100+Cd100. The staining results (Figure 2a,c) indicated that O_2_^−^ and H_2_O_2_ mainly increased in the leaf veins of *S. nigrum*.

### 2.3. Transcriptomic and Proteomic Analysis Overview

**Figure 3:**
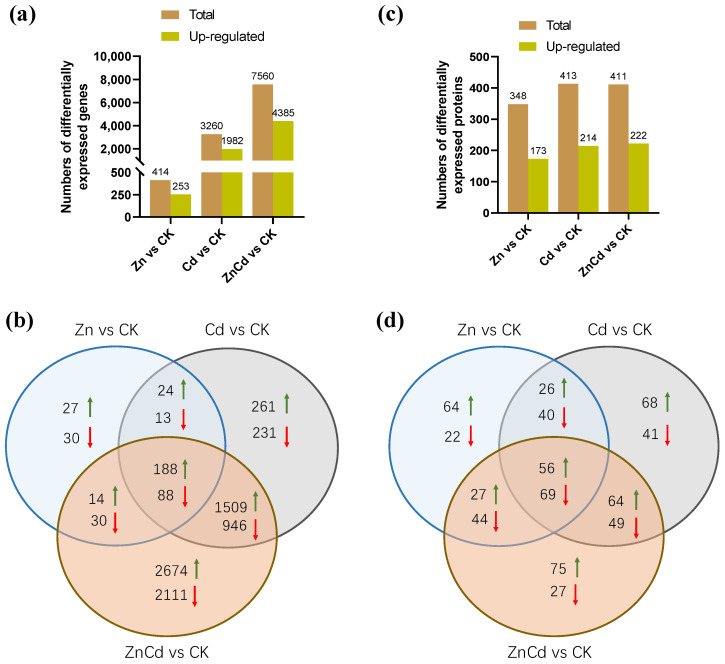
The numbers of differentially expressed genes (**a**,**b**) and differentially expressed proteins (**c**,**d**) in leaves of *S. nigrum* by transcriptome and proteome. Plants were exposed to a complete Hoagland solution (CK) or with 100 μmol·L^−1^ Zn (Zn), 100 μmol·L^−1^ Cd (Cd), and 100 μmol·L^−1^ Zn +100 μmol·L^−1^ Cd (ZnCd) for 10 days. Rising green arrow shows increase, and falling red arrow shows decrease in significant differential expression between sample set (Zn vs. CK, Cd vs. CK, and ZnCd vs. CK).

In the transcriptome of *S. nigrum*, 61,729 non-redundant transcripts were annotated, and a total of 8146 DEGs were screened. Most of the DEGs were in Cd100 and Zn100+Cd100 (Figure 3a), and the number of upregulated DEGs was greater than that of downregulated DEGs. As shown in our Venn diagrams (Figure 3b), there were 188 upregulated and 88 downregulated DEGs in the three treatments, and 1509 upregulated and 946 downregulated DEGs were in both Cd100 and Zn100+Cd100.

Proteome MS/MS data were searched against the *S. nigrum* transcriptome, and a total of 672 DEPs were screened. Almost half of the DEPs were upregulated in each treatment (Figure 3c). There were 56 upregulated and 69 downregulated DEPs in the three treatments, and 64 upregulated and 49 downregulated DEPs were in both Cd100 and Zn100+Cd100 (Figure 3d).

### 2.4. Expression Characteristic of Transporter Genes in Leaves of S. nigrum under Zn and Cd

**Figure 4:**
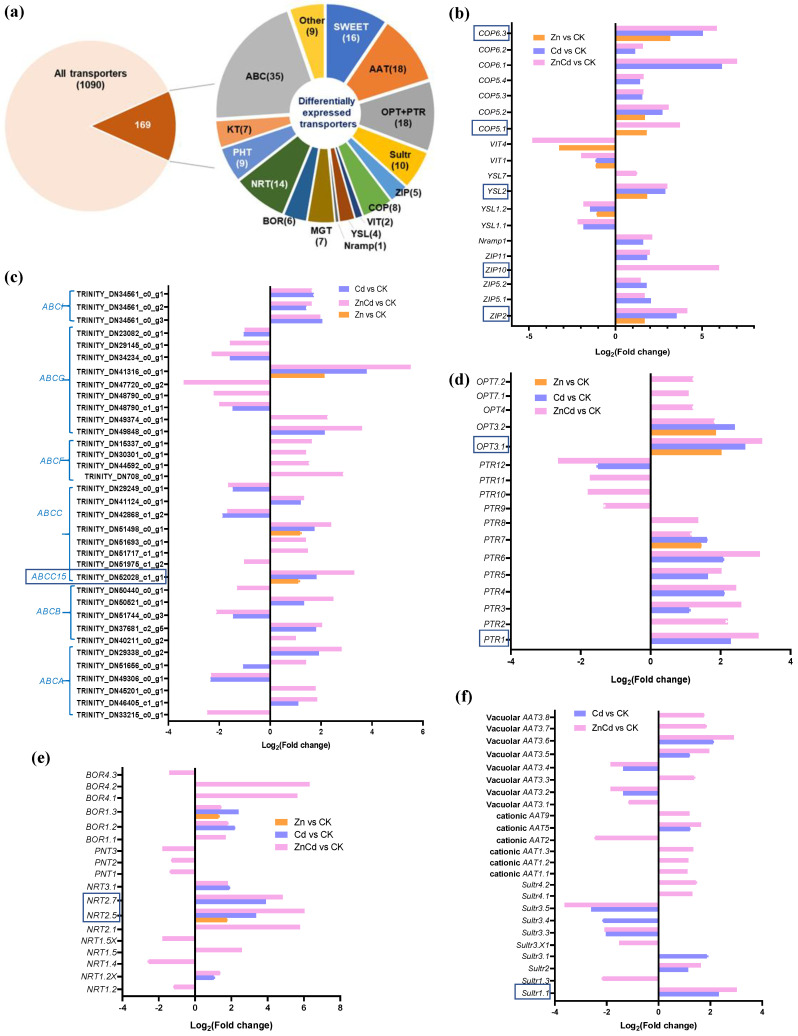
Identification and gene expression levels of significantly differentially expressed transporters in leaves of *S. nigrum* by transcriptome. Proportions of the identified transporters (**a**). Gene expression level of metal transporters (**b**); ABC transporters (**c**); peptide transporters (**d**); nitrate, phosphate, and boron transporters (**e**); and sulfate and amino acid transporters (**f**). The boxed transporter genes were then verified by qRT-PCR. Plant was exposed to a complete Hoagland solution (CK) or with 100 μmol·L^−1^ Zn (Zn), 100 μmol·L^−1^ Cd (Cd), and 100 μmol·L^−1^ Zn+100 μmol·L^−1^ Cd (ZnCd) for 10 days. Expression levels of transporters shown use Log_2_ (fold change) between sample sets (Zn vs. CK, Cd vs. CK, and ZnCd vs. CK). *ABC* (*A*, *B*, *C*, *F*, *G*, *I*): ATP-biding cassette transporter six subfamilies; *Sultr*, sulfate transporter; *AAT*, amino acid transporter; *ZIP*, zinc transporter; *COP*, copper transporter; *Nramp*, natural resistance associated macrophage protein; *YSL*, metal–nicotianamine transporter; *VIT*, vacuolar iron transporter; *MGT*, magnesium transporter; *PTR*, peptide transporter; *OPT*, oligopeptide transporter; *NRT*, nitrate transporter; *PNT*, peptide/nitrate transporter; *BOR*, boron transporter; *KT*, potassium transporter; *PHT*, phosphate transporter; *SWEET*, bidirectional sugar transporter.

A total of 1090 non-redundant transporters were identified from the transcriptome of *S. nigrum* leaves, with 169 transporter genes identified as DEGs in Zn and Cd treatments (Figure 4a). Five Zn transporters (*ZIPs*), a natural resistance-associated macrophage protein gene (*Nramp1*), a metal–nicotianamine transporter gene (*YSL2*), and six copper transporter genes (*COPs*) were upregulated in Cd100 and Zn100+Cd100, and *ZIP2*, *YSL2*, *COP5.1*, *COP5.2,* and *COP6.3* were also upregulated in Zn100. However, *YSL1.1*, *YSL1.2*, and two vacuolar iron transporter genes (*VIT1* and *VIT4*) were downregulated in Zn100+Cd100 (Figure 4b). Thirty-five DEGs were identified as ATP-binding cassette transporters (*ABCs*), which accounted for one-fifth of the differentially expressed transporter genes. The *ABCs* included the six subfamilies of *ABCA*, *ABCB*, *ABCC*, *ABCF*, *ABCG*, and *ABCI*, and all differentially expressed *ABCs* were regulated in Zn100+Cd100. One of the *ABCG* genes had the highest upregulation in Zn100+Cd100, and three ABCI genes were upregulated in both Cd100 and Zn100+Cd100 (Figure 4c). Eight peptide transporter genes (*PTRs*) and five oligopeptide transporter genes (*OPTs*) were upregulated in Zn100+Cd100, with most also upregulated in Cd100. The genes *PTR7*, *OPT3.1*, and *OPT3.2* were also upregulated in Zn100 (Figure 4d). Six nitrate transporter genes (*NRTs*) and five boron transporter genes (*BORs*) were upregulated in Zn100+Cd100. Four of the *NRTs* and two of the *BORs* were also upregulated in Cd100, and *NRT2.5* and *BOR1.3* were also upregulated in Zn100 (Figure 4e). Five vacuolar amino acid transporter genes (*AATs*), five cationic *AATs*, and five sulfate transporter genes (*Sultrs*) were upregulated in Zn100+Cd100; however, none responded in Zn100 (Figure 4f). In addition, DEGs included sixteen sugar-transporter genes (*SWEETs*), nine phosphate-transporter genes (*PHTs*), seven magnesium-transporter gene (*MGTs*), seven potassium-transporter genes (*KTs*), and nine genes of other transporters (Figure 4a).

**Figure 5:**
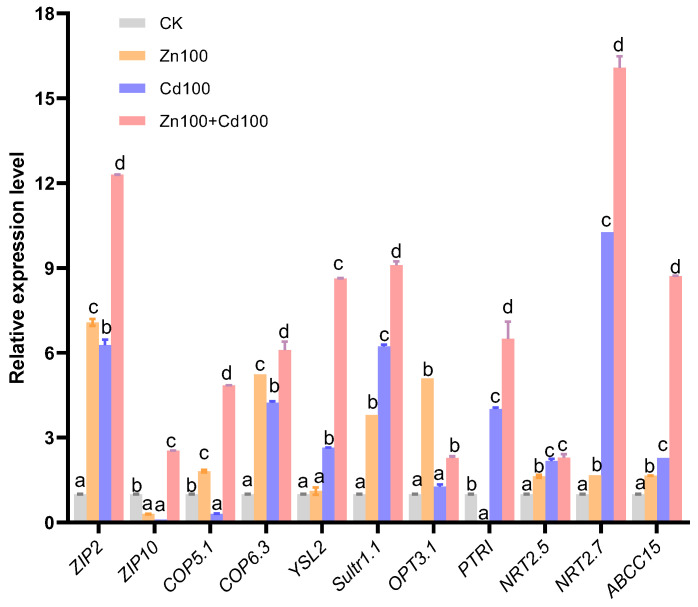
Relative gene expression level of transporters in leaves of *S. nigrum* by qRT-PCR. Plant was exposed to a complete Hoagland solution (CK) or with 100 μmol·L^−1^ Zn (Zn100), 100 μmol·L^−1^ Cd (Cd100), and 100 μmol·L^−1^ Zn+100 μmol·L^−1^ Cd (Zn100+Cd100) for 10 days. Relative expression level of genes denoted by different letters refer to the significant differences (*p* < 0.05, Duncan’s test).

Eleven transporter genes were selected from the DEGs of the *S. nigrum* transcriptome (the boxed transporter genes in Figure 4b–f), and the relative expression levels were verified by qRT-PCR. As shown in Figure 5, the gene expression levels of all transporters selected were consistent with the results of the transcriptome in Zn100+Cd100, and the expression levels of *ZIP2*, *COP6.3*, *Sultr1.1*, *NRT2.5*, *NRT2.7*, and *ABCC15* were consistent with the results of the transcriptome in the three treatments.

### 2.5. Differentially Expressed Genes Involved in Antioxidative Defense

Seventeen DEGs were involved in antioxidative defense (Supplementary Figure S1a), including four metallothionein genes (*MTs*), a sulfite reductase gene (*SiR1*), four catalase genes (*CATs*), two ferredoxin–NADP reductase genes (*FNR*), two L-ascorbate peroxidase genes (*APXs*), two peroxidase genes (*PODs*), a Fe superoxidase dismutase gene (*FeSOD*), and a Cu and Zn superoxidase dismutase gene (*Cu*/*ZnSOD*). Most antioxidant protein genes were upregulated, except for *MT2a*, *SiR1*, and *Cu*/*ZnSOD*, which were downregulated in both Cd100 and Zn100+Cd100; and three CAT genes, which were downregulated in Zn100. Relative expression levels of four MT genes (*MT2a*, *MT2b*, *MT2bX1*, and *MT2c*) were verified by qRT-PCR (Supplementary Figure S1b). The *MTs* upregulated in the transcriptome were confirmed by the expression levels in qRT-PCR; however, the expression of *MT2a* was not consistent in Zn100+Cd100. In Cd100 and Zn100+Cd100, MT2c was the most upregulated gene in both transcriptome and qRT-PCR analyses.

### 2.6. Differentially Expressed Genes and Proteins Involved in GSH and Malate Metabolic Pathways

Twenty cysteine-rich receptor-like protein kinase genes (*GRKs*), nine cysteine proteinase precursor genes (*CysPs*), and two cysteine synthase genes (*CysSs*) were upregulated in Zn100+Cd100 according to the transcriptome of *S. nigrum* leaves. Some of those genes also were upregulated in Cd100, whereas *CysP1* and *CysP5* were downregulated in Zn100+Cd100 (Supplementary Figure S2a). Of the glutathione S-transferases (*GSTs*), 39 of 40 DEGs were upregulated in Zn100+Cd100, with 14 also upregulated in Cd100 (Supplementary Figure S2b).

**Figure 6:**
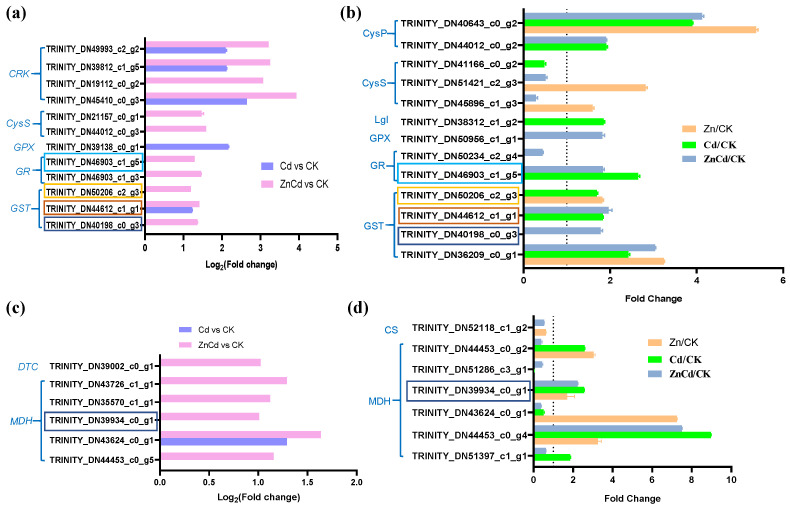
Expression levels of DEGs and DEPs involved in glutathione (**a**,**b**) and malate (**c**,**d**) metabolism in leaves of *S. nigrum* by transcriptome and proteome. The boxes with the same color are the same genes. Plant was exposed to a complete Hoagland solution (CK) or with 100 μmol·L^−1^ Zn (Zn), 100 μmol·L^−1^ Cd (Cd), and 100 μmol·L^−1^ Zn+100 μmol·L^−1^ Cd (ZnCd) for 10 days. Expression level of gene by transcriptome was shown using Log_2_ (fold change) between sample sets (Zn vs. CK, Cd vs. CK, and ZnCd vs. CK). Expression level of protein by proteome was shown using a fold change (*p* < 0.05, Student’s *t*-test) between sample sets (Zn/CK, Cd/CK, and ZnCd/CK). GRK, cysteine-rich receptor-like protein kinase; CysS, cysteine synthase; GPX, glutathione peroxidase; GR, glutathione reductase; GST, glutathione S-transferase; CysP, cysteine proteinase precursor; Lgl, lactoylglutathione lyase; MDH, malate dehydrogenase; DTC, dicarboxylate/tricarboxylate transporter; CS, ATP-citrate synthase.

Notably, three *GSTs* and a glutathione reductase (*GR*) were identified in both the transcriptome and proteome in leaves of *S. nigrum*, and their expression was most upregulated in Zn100+Cd100 (Figure 6a,b). Five DEGs and six DEPs were identified as mitochondrial malate dehydrogenases (MDHs) in S. nigrum leaves, and one MDH was identified as being upregulated in both the transcriptome and proteome in Zn100+Cd100 (Figure 6c,d).

### 2.7. Differentially Expressed Proteins Involved in Metabolism of Chlorophyll and ATP

**Figure 7:**
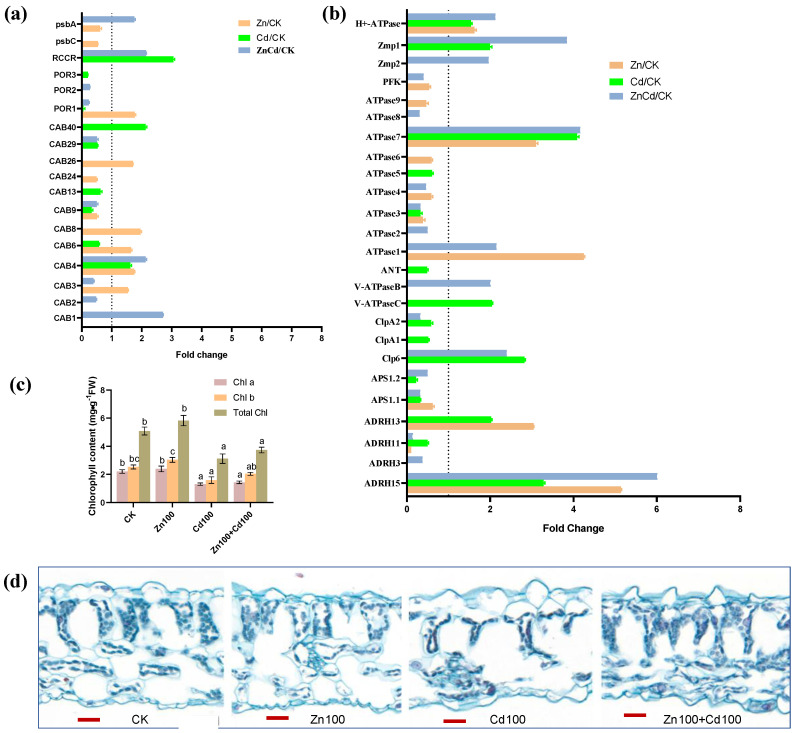
Expression levels of DEPs involved in chlorophyll (**a**) and ATP metabolism (**b**), chlorophyll content (**c**), and cytochemical characteristics (**d**) in leaves of *S. nigrum*. Plant was exposed to a complete Hoagland solution (CK) or with 100 μmol·L^−1^ Zn (Zn), 100 μmol·L^−1^ Cd (Cd), and 100 μmol·L^−1^ Zn+100 μmol·L^−1^ Cd (ZnCd) for 10 days. Expression level of protein was shown using a fold change (*p* < 0.05, Student’s *t*-test) between sample sets (Zn/CK, Cd/CK, and ZnCd/CK). Chlorophyll (Chl) contents denoted by different letters refer to the significant differences (*p* < 0.05, Duncan’s test). Paraffin-section experiments were repeated at least three times with similar results; bar, 20 μm. psbA, photosystem I P700 chlorophyll apoprotein; psbC, photosystem II CP43 chlorophyll apoprotein; RCCR, red chlorophyll catabolite reductase; POR, protochlorophyllide reductase; CAB, chlorophyll *a*/*b* binding protein; H^+^-ATPase, plasma membrane H^+^-ATPase; Zmp, ATP-dependent zinc metalloprotease; PFK, ATP-dependent 6-phosphofructokinase; ANT, ADP/ATP translocator; V-ATPase, vacuolar-type ATPase; ClpP, ATP-dependent Clp protease; ASP, ATP sulfurylase; ADRH, ATP-dependent RNA helicase.

As shown in Figure 7a, 12 DEPs were identified as chlorophyll a-b binding proteins (CABs). Five CABs were upregulated in Zn100, CAB1 was only upregulated in Zn100+Cd100, CAB40 was only upregulated in Cd100, and CAB4 was upregulated in the three treatments. A protochlorophyllide reductase (POR1), a chlorophyll apoprotein (psbA), and a red chlorophyll catabolite reductase (RCCR) were upregulated in Zn100, Zn100+Cd100, and Cd100, respectively. Chlorophyll (Chl) content, including Chl *a*, Chl *b*, and total Chl, was not significantly affected in the leaves of *S. nigrum* under Zn treatment for 10 d, but content decreased significantly in Cd100 and Zn100+Cd100 (Figure 7b).

Twenty-five DEPs were involved in ATP metabolism (Figure 7c), including the plasma membrane H^+^-ATPase, two vacuolar-type ATPases (V-ATPases), and nine ATPases. The H^+^-ATPase and ATPase 7 were upregulated in the three treatments, and ATPase1 had the highest upregulation in Zn100, followed by that in Zn100+Cd100. The V-ATPaseB and V-ATPaseC were upregulated in Zn100+Cd100 and Cd100, respectively. In addition, two ATP-dependent zinc metalloproteases (Zmps), an ATP-dependent Clp proteases (Clp6), and an ATP-dependent RNA helicase (ADRH15) had the highest upregulation in Zn100+Cd100, followed by that in Zn100 or Cd100. However, an ATP-dependent 6-phosphofructokinase, an ADP/ATP translocator, and two ATP sulfurylases were downregulated in Cd100 or Zn100. 

From our microscopic observation, we observed that the numbers of chloroplasts decreased significantly in Cd100; however, the numbers of chloroplasts and mesophyll cells increased sharply in leaves of *S. nigrum* in Zn100+Cd100 (Figure 7d).

## 3. Discussion

### 3.1. Zn Supplement Increases Cd Accumulation in Shoots of S. nigrum

Compared with separate Zn or Cd treatment, Zn supplement with Cd significantly increased Cd accumulation but decreased Zn accumulation in the shoots of *S. nigrum* (Figure 1c,d). The results are similar to Cd accumulation in the petioles and leaves of *Potentilla griffithii* Hook, a Zn/Cd hyperaccumulator, under Zn and Cd compound treatment [4]. The authors proposed that proportions of Zn and Cd in hydroponic culture determined the levels of Zn and Cd accumulation in *P. griffithii* [4,5]. Moreover, the increase in Cd content in xylem saps was consistent with that in the stems and leaves of *S. nigrum* in Cd100, indicating that Cd was transported through xylem in *S. nigrum*. The Zn contents in xylem and phloem saps were consistent with those in the stems and leaves of *S. nigrum*, indicating that Zn was transported by both the xylem and phloem (Figure 1a,b).

### 3.2. Zn Supplement Decreases the Production of ROS in Leaves of S. nigrum 

Zn at a low concentration (below 100 μmol·L^−1^ Zn) can improve plant growth and inhibit ROS generation [31,32]. In this study, the production of O_2_^−^ and H_2_O_2_ in the leaves of *S. nigrum* in Zn100 decreased compared with that in the control, and O_2_^−^ and H_2_O_2_ also decreased in Zn100+Cd100 compared with Cd100 (Figure 2a,d). The results indicated that *S. nigrum* benefited from the 100 μmol·L^−1^ Zn treatment, although the Zn concentration exceeded that suitable for most plants. The results are also consistent with the production of O_2_^−^ and H_2_O_2_ in rice leaves under Zn and Cd combined treatments [7,8].

### 3.3. Zn Supplement Promotes Cd Transport and Sequestration in Leaves of S. nigrum

Most Cd hyperaccumulators can efficiently transport Cd to aboveground parts using various transporters, and some metal transporters, in particular, are noted for their capacity to load Cd into the xylem and increase the Cd concentration in plant shoots [26,33]. Nramp family genes, which are involved in the transport of a wide range of divalent cations, are upregulated during the *S. nigrum* response to Cd or Zn treatment [18,22,34]. The gene *SaNramp6* is upregulated in the roots of the hyperaccumulator *S. alfredii* exposed to Cd, and the expression of *SaNramp6* in *A. thaliana* increases the net Cd^2+^ fluxes in shoots under Cd treatment [33]. The ZIPs can transport a variety of cations, including those of Cd, Zn, Mn, and Fe [35]. However, transporters can also have opposite functions; for example, knockout of *OsZIP7* leads to an increase in Cd accumulation in rice roots [36]. The COP transporters are important in maintaining Cu homeostasis in plants under different stress conditions [37]. In this study, three *COPs*, *ZIP2*, *Nramp1*, and *YSL2* had the highest upregulation in Zn100+Cd100, followed by that in Cd100. Notably, *YSL2* and *ZIP2* in the leaves of *S. nigrum* were significantly upregulated in the three treatments in both transcriptome (Figure 4b) and qRT-PCR results (Figure 5). Therefore, *S. nigrum* can uptake and transport Cd and Zn by cooperation among transporters of YSL2, ZIPs, and Nramp1. Vacuolar iron transporter (VIT) is an Fe transporter, which can transport cytoplasmic Fe ions into vacuoles [38]. In *S. nigrum* under Cd treatment, Fe utilization decreased, and plants exhibit an Fe deficiency signal [18,25], which could explain why the two VIT genes were downregulated in the present study.

In this study, approximately 16% of transporters in *S. nigrum* leaves were significantly regulated by Zn and Cd treatment, with 35 of the transporter genes identified as ABC transporters. The ABC transporters have crucial roles in the metabolism of plant secondary metabolites and response to environmental stress [39,40]. On the thylakoid membrane of rice, OsABCI7 can regulate intracellular ROS homeostasis and maintain thylakoid membrane stability [40]. In Arabidopsis, *AtABCI10* and *AtABCI11* are significantly induced by Fe deficiency and regulate chloroplast biogenesis and metal homeostasis [41]. In addition, OsABCG43 is important in the sequestration of Cd into subcellular organelles, thereby reducing Cd toxicity to rice [14]. The transporter AtABCG40 on plasma membrane acts as a pump to exclude lead and other toxic compounds from the cytoplasm [42], and OsABCC1 is upregulated by arsenic and sequesters it in the vacuoles of nodes of rice [43]. In this study, there were six DEGs of ABC subfamilies, with three ABCI family genes upregulated in Cd100 and Zn100+Cd100, and an ABCG (TRINITY_DN41316_c01_g1) and two ABCC family genes (including *ABCC15*) upregulated in the three treatments in the order Zn100+Cd100 > Cd100 > Zn100 (Figure 4c). Therefore, the DEGs of ABCG and ABCCs were induced by Cd and Zn in the present study, which could have vital roles in Cd sequestration in vacuoles and xylem transport in *S. nigrum*.

Furthermore, all OPT and 8 of 12 PTR DEGs were upregulated in Zn100+Cd100, and almost half were also upregulated in Cd100 (Figure 4d). Peptides complexed with heavy metals can be transported to plant vacuoles or cell walls by PTRs and OPTs [10,44]. AtOPT6 is a low-affinity GSH transporter that loads the phloem in Arabidopsis leaves [45], and OsOPT3 has a key role in long-distance GSH transport in rice [44,46]. However, *TcOPT3* is highly induced by Fe deficiency in *N. caerulescens* [47]. Therefore, it is hypothesized that the upregulation of PTR and OPT genes in *S. nigrum* leaves increases the complexation of GSH and Cd, with complexes then entering vacuoles or cell walls to sequester Cd.

Amino acids are essential for peptide or protein biosynthesis in plants, and amino acids stored in vacuoles are exported to the cytosol mainly for metabolic and signaling roles [48]. Amino acid transport systems are not highly specific for single amino acids in rice, *Populus trichocarpa*, and *Solanum tuberosum* [48,49,50]. Five AAT genes in rice have vital roles in C and N metabolism and distribution [49], and excess Zn increases the expression of Arabidopsis *AtNRT1.1* to promote nitrate absorption and Zn transport [51]. In addition, 50 μM Cd promotes NH_4_^+^ absorption by upregulating the gene expressions of NH_4_^+^ transporters in *S. nigrum* [19]. In the present study, most AAT and NRT DEGs were upregulated in the order Zn100+Cd100 > Cd100 in *S. nigrum* leaves (Figure 4e,f), and with Zn supplementation, we noted increased N metabolism in leaves and reduced Cd toxicity to *S. nigrum*.

### 3.4. Zn Supplement Decreased Cd Toxicity in Leaves of S. nigrum 

Vacuolar sequestration is a good strategy to safely store metal ions and detoxify toxic metals [9]. In the leaves of *P. griffithii*, protoplasts contained 94% and 70% of the total Zn and Cd, respectively, and more than 90% of the Cd and Zn in the protoplasts was localized in vacuoles [4]. Cd in the roots and fresh leaves of *S. nigrum* is mainly bound to thiol ligands [24,27], and the levels of cysteine and GSH increase significantly in the roots of *S. nigrum* under Cd treatment [24]. In the present study, the DEGs involved in cysteine and GSH metabolism included 11 *CysPs*, 20 *CRKs*, and 40 *GSTs*, most of which were upregulated in Zn100+Cd100; and one-third of those DEGs were also upregulated in Cd100 (Supplementary Figure S2a,b).

Both the genes and proteins of three GSTs and a GR were upregulated in the leaves of *S. nigrum* in Zn100+Cd100 or Cd100, whereas the expression of other genes and proteins, including GPX, CysS, and CysP, was not inconsistent (Figure 6a,b). The GSTs can transport GSH-Cd into a vacuole by tonoplast ABC transporters [52]. The GSH is oxidized to yield oxidized glutathione (GSSG) by GPX, which is then reduced back to GSH by GR [9]. Activities of GST, GR, and GPX control the overall cellular redox status in *Urtica dioica* response to Cd [53]. Our results indicated that Zn supplement increased GSH synthesis and transport in the leaves of *S. nigrum*. However, the level of GSH depends on the availability of the substrate cysteine. In this study, DEGs of SULTR transporter, cysteine synthase, and cysteine proteinase precursor were upregulated in Zn100+Cd100 (Figure 4f and Supplementary Figure S2a,b). The DEGs could be used to produce metal–thiol complexes, such as those with GSH, PCs, and MTs. Notably, MTs protect cells against metal-caused oxidative stress by chelating excess metals via cysteine thiol groups [9,11,54]. Zn induces an increase in MT transcripts in both the roots and shoots of *S. nigrum* [54,55]. There were two MT2b genes upregulated in Zn100 according to both transcriptome and qRT-PCR results, and, similarly, an MT2c gene was upregulated in Cd100 and Zn100+Cd100 (Supplementary Figure S1a,b). The results indicated that MT genes mainly induced by Zn100 could chelate Zn and eliminate ROS, thereby explaining the decreases in O_2_^−^ and H_2_O_2_ in the leaves of *S. nigrum* exposed to Zn treatment (Figure 2a–d). 

Several DEGs, including one *FeSOD*, two *PODs*, two *APXs*, and two *FNRs* involved in antioxidant protection, were upregulated in Zn100+Cd100 or Cd100, and three *CATs* were downregulated in Zn100 (Supplementary Figure S1a,b). It was hypothesized that Cd-induced ROS in the leaves of *S. nigrum* would cause the expression of DEGs involved in antioxidant protection to increase, and *MTs* upregulated in Zn100 would scavenge ROS. Therefore, the levels of ROS were moderated in Zn100+Cd100.

### 3.5. Zn Promotes Chlorophyll and ATP Biosynthesis in Leaves of S. nigrum 

Cd accumulation in leaves of *S. nigrum* damages the cell organelles and causes swollen chloroplasts and deformed cell walls [18]. Chloroplasts are the major sink for Fe in leaves, and Fe is also an essential cofactor for chlorophyll biosynthesis enzymes, with low Fe leading to a decrease in chlorophyll synthesis [32,56]. Five DEGs of CABs and POR1 were upregulated in Zn100 in *S. nigrum*, and CAB1 and chlorophyll apoprotein (psbA) were also upregulated in Zn100+Cd100 (Figure 7a). Chlorophyll is bound to different chlorophyll-binding proteins, which then become the core complexes of the two photosystems [57]. Chlorophyll biosynthesis is regulated by the expression of CABs and psbAs in *Camellia sinensis* [58] and Arabidopsis [57]. In the leaves of *N. caerulescens*, exogenous Zn increases chlorophyll biosynthesis by promoting Fe uptake and accumulation [59]. The POR catalyzes the light-dependent reduction of protochlorophyllide to chlorophyll [60], and RCCR catalyzes key steps in chlorophyll degradation [61]. Cd depresses the activities of Rubisco and PSII in Arabidopsis [57]. In this study, POR1 was upregulated in Zn100, and RCCR was upregulated in Cd100 and Zn100+Cd100 (Figure 7a), which could explain the increase in numbers of chloroplasts and mesophyll cells in *S. nigrum* in Zn100+Cd100 compared with that in Cd100 (Figure 7d). 

Zn–malate complexes are the main accumulation forms in the hyperaccumulators *A. halleri* [15] and *N. caerulescens* [14]. The MDHs catalyze the interconversion of malate and oxaloacetate coupled with the electron transport chain (ETC) of reduction or oxidation of NAD(P)^+^/NAD(P)H in mitochondria and chloroplasts [62]. Malate and oxaloacetate are exported and imported into mitochondria and chloroplasts via a dicarboxylate/tricarboxylate transporter (DTC), and then the ETC of oxidation of mitochondrion NADH can activate signaling systems to modulate energy metabolism [62]. In the present study, DEGs of five MDHs and a DTC were upregulated in Zn100+Cd100, and three MDH proteins were upregulated in Zn100 (Figure 6c,d). The results indicated that Zn can improve energy metabolism by regulating the NAD(P)^+^/NAD(P)H system in mitochondria.

The V-ATPases have vital roles in intracellular acidic compartments [63], and the plasma membrane H^+^-ATPase modulates stomatal closure in Arabidopsis under stress [64]. In this study, two ATP synthases and two ADRHs were upregulated in Zn100 and Zn100+Cd100; V-ATPase C and B were upregulated in Cd100 and Zn100+Cd100, respectively; and the plasma membrane H^+^-ATPase was upregulated in the three treatments (Figure 7c) and might benefit the response of *S. nigrum* to Cd stress. The ATP-dependent Clp and Zmp are major proteases in chloroplasts of Arabidopsis [65]. A Clp is involved in Fe homeostasis in Arabidopsis leaves, with the loss of *Clp* resulting in a decrease in chloroplasts [66], and Zmp has critical roles in the biogenesis of thylakoid membranes [65]. In this study, Clp6, Zmp1, and Zmp2 were upregulated in Zn100+Cd100 (Figure 7c), which can explain the increase in the numbers of chloroplasts and mesophyll cells in leaves of *S. nigrum*. Cytochemical observations support the conclusion (Figure 7d).

## 4. Materials and Methods

### 4.1. Plant Material and Treatment

The wild seeds of *S. nigrum* were collected from Huaguoshan mine in Luoyang, China (39°19′ N, 111°53′ E). Seeds were sown in vermiculite for 15 d, and then the seedlings with similar growth were cultured in 2.5 L plastic vessels with Hoagland full solution (1 mM KNO_3_, 1 mM KH_2_PO_4_, 1 mM MgSO_4_, 1 mM Ca(NO_3_)_2_, 46 μM H_3_BO_3_, 9 μM MnCl_2_, 0.76 μM ZnSO_4_, 0.32 μM CuSO_4_, 0.11 μM H_2_MoO_4_, and 20 μM Fe-EDTA) under controlled conditions (14-hour day length with 400 μmol m^−2^ s^−1^ photosynthetically active radiation). Seedings with four leaves were cultivated in Hoagland full solution with the following treatment: control (CK), 100 μmol·L^−1^ Zn (Zn100), 100 μmol·L^−1^ Cd (Cd100), and 100 μmol·L^−1^ Zn and 100 μmol·L^−1^ Cd (Zn100+Cd100). Elements for treatment were applied as ZnSO_4_·7H_2_O and CdCl_2_. After Zn and Cd treatment for 10 d, leaves, stems, and xylem and phloem saps were collected and analyzed for metal contents. The second youngest leaves were cut for transcriptome sequencing and proteome analysis, quantitative real-time PCR (qRT-PCR), detection of H_2_O_2_ and O_2_^−^, determination of chlorophyll content, and paraffin sectioning.

### 4.2. Preparation of Phloem and Xylem Saps

To collect *S. nigrum* phloem saps, the surfaces of the petioles of the second youngest leaves were cut with a razor blade. After excluding the first drops, the exuded drops of phloem saps were collected using 100 μL pipettes, with the pH of exuded drops approximately 8.0 [67]. To collect xylem saps, stems were cut with a razor blade approximately 1.5 cm above the roots. After excluding the first drop, drops of xylem sap were collected using 100 μL pipettes, with the pH of exuded drops being 6.0–6.5 [67]. Phloem and xylem saps were stored at 4 °C until analysis of Zn and Cd contents.

### 4.3. Determination of Zn and Cd Content

Leaves and stems of *S. nigrum* were collected, washed with distilled water, and then dried in an air circulation oven at 70 °C. Dried samples, approximately 0.2 g, were digested by HNO_3_:HClO_4_ (*v*/*v*, 87:13), following the procedure described by Zhang et al. [68]. Phloem and xylem saps were diluted twice with 10% HNO3 before the determination of metal contents. An ICP-OES (Optima 8000, PerkinElmer, Waltgam, MA,USA) was used to analyze *S. nigrum* contents of Zn and Cd. The certified reference materials (CRMs) are liquid standards of Zn and Cd provided by National Research Center for Certified Reference Materials (Beijing, China). Concentrations of calibration solutions were as follows: Zn^2+^ (0, 1, 2, 3, 4, and 5 mg/L) and Cd^2+^ (0, 0.1, 0.2, 0.3, 0.4, and 0.5 mg/L). The instrument software uses linear calibration to determine the concentrations of Zn and Cd in the digested *S. nigrum* samples. These concentrations were used together with the sample weights and volumes to determine the concentrations of Zn and Cd in *S. nigrum*.

### 4.4. Hydrogen Peroxide (H_2_O_2_) and Superoxide Anion (O_2_^−^) Localization In Situ

The production of H_2_O_2_ and O_2_^−^ in leaves of *S. nigrum* was detected with 3,3′-diaminobenzidine (DAB) and nitro-blue tetrazolium chloride (NBT) staining, respectively, following the procedures described by Zhang et al., with some modifications [69]. The second youngest leaves with petioles were incubated in a 1.0 mg·mL^−1^ solution of DAB (pH 3.8) for H_2_O_2_ detection, or in 0.1% NBT in 50 mM of K-phosphate buffer containing 10 mM sodium azide (pH 6.4) for O_2_^−^ detection. After incubation at room temperature for 4 h in the dark, leaves were vacuum infiltrated for 10 min and then bleached in boiling ethanol. Images were captured with a Nikon D7100 digital camera (Nikon, Ayutthaya, Thailand).

### 4.5. Determination of H_2_O_2_ and O_2_^−^ in Leaf Extract

The content of H_2_O_2_ was determined by monitoring the absorbance at 415 nm of the titanium–peroxide complex, and the O_2_^−^ production rate was measured by monitoring the absorbance at 530 nm of the nitrite formation from hydroxylamine hydrochloride reacting with O_2_^−^. The two determination methods were described by Deng et al. [24].

### 4.6. Microscopic Observation of Mesophyll Cells

Leaf pieces (1.0 to 2.0 mm^2^) were cut from the second youngest leaves and immersed in FAA fixative solution (Gefan Biotech, Shanghai, China). Paraffin sections were prepared according to the method described by Maniou et al. [70] and stained following Johansen’s safranin and fast green protocol [71].

### 4.7. Determination of Chlorophyll Content

Fresh samples, approximately 1.0 g, from the second youngest leaves were extracted in 10 mL of 80% acetone, and chlorophyll content was determined according to the method described by Zhang et al. [68].

### 4.8. Transcriptome Sequencing

Leaf RNA was extracted using a FastPure Plant Total RNA Isolation kit (Vazyme, Nanjing, China) and then reverse transcribed using a Hifair TM II Ist Strand cDNA Synthesis kit (Yeasen, Shanghai, China), according to instructions of the manufacturers. After the library passed inspection, high-throughput sequencing was performed using a HiSeq sequencing platform (Genepioneer Biotech, Nanjing, China) according to the method of Zhang et al. [72]. After raw data were filtered, 89.31 Gb of clean data was obtained (Supplementary Table S1). Sequenced reads were assembled with Trinity software (Trinity Release v2.13.2, 4 September 2021) to obtain 272,507 transcripts and 114,602 unigenes (26442 above 1 kb). Using NCBI blast, *S. nigrum* unigene sequences were compared with NCBI non-redundant protein sequences (NR), Swiss-Prot protein sequence (Swiss-Prot), Gene Ontology (GO), Protein family (Pfam), and Kyoto Encyclopedia of Genes and Genome (KEGG) databases to obtain unigene annotation information [18]. Differential expression analysis of two samples was performed using the EBSeq R package (https://bioconductor.org/packages/release/bioc/html/EBSeq.html, accessed on 22 September 2022). Significance *p*-values (*p* < 0.05) were corrected with the Benjamin–Hochberg method [18], and |log_2_(fold change)| > 1 was set as the threshold for significant differential expression between Zn100 and CK, between Cd100 and CK, and between Zn100+Cd100 and CK. Fold change represented the ratio of gene expression levels between treatment and control samples. When the log_2_(fold change) value was positive, a differentially expressed gene (DEG) was upregulated, whereas when the value was negative, a DEG was downregulated.

### 4.9. Quantitative Real-Time PCR Analysis (qRT-PCR)

Leaf RNA was extracted and reverse-transcribed using the same kits and procedures as those in the transcriptome analysis. Primers were designed online (https://sg.idtdna.com/PrimerQuest/Home/Index, accessed on 20 November 2022) according to the cds from the *S. nigrum* transcriptome (Supplementary Table S2). A Bio-Rad CFX System and Hifair III One Step RT-qPCR SYBR Green Kit (Yeasen) were used for qRT-PCR analysis. Relative expression of genes was normalized by the 2^−ΔΔCt^ method, and *S. nigrum EF1a* (GenBank AY574951.1) was used as the internal reference gene.

### 4.10. Proteome Analysis

Leaf protein was extracted and determined according to the method of Zhang et al. [72]. Proteins, approximately 500 μg, were dissolved in lysis solution (50 mM Tris-HCl, 8.0 M urea, and 1.0 M dithiothreitol, pH 8.0). Subsequently, protein samples were digested with trypsin, and proteomic analysis was performed using liquid chromatography–tandem mass spectrometry (LC-MS/MS)-based label-free quantification according to the method of Duan et al. [73]. The MS/MS data were searched against the S. nigrum transcriptome using Proteome Discoverer v2.1 software (Thermo Fisher Scientific, Waltham, MA, USA). Significantly differentially expressed proteins (DEPs) were those with a 1.5-fold (up) or 0.67-fold (down) change and *p* < 0.05 (Student’s *t*-test) between Zn100 and CK, between Cd100 and CK, and between Zn100+Cd100 and CK.

### 4.11. Statistical Analyses

Values are presented as the means ± standard errors (*n*= 3), and means denoted by different letters are significantly different (*p* < 0.05, Duncan’s test). Data were analyzed using SPSS 22.0, and figures were prepared with GraphPad Prism 9.0. The stained pictures were repeated at least five times, with similar results.

## 5. Conclusions

**Figure 8:**
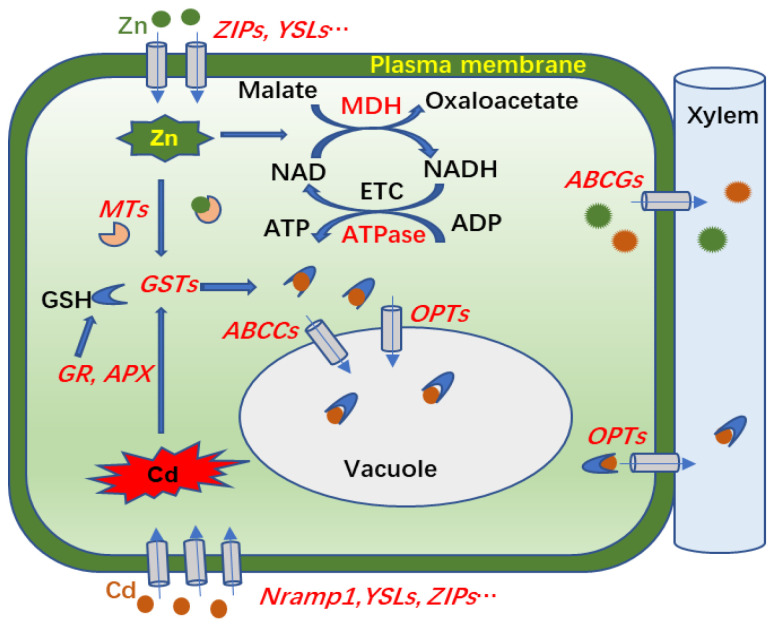
Molecular mechanism involved in transport and accumulation of Cd in leaves of *S. nigrum* exposed to Zn and Cd. Magenta and green pellets indicate Cd and Zn, respectively; and the genes or proteins in red font represent those upregulated by Cd or Zn in leaves of *S. nigrum*. Cd or Zn enters into leaf cells by plasma membrane transporters of *Nramp1*, *YSLs*, *ZIPs*, etc.; *MTs* and *GSTs* in cells are induced for antioxidant protection or chelation with excess metal ions; and then Cd-GSH complexes are transported to vacuoles for sequestration, or to cell walls for xylem transport by ABCs and OPTs. In addition, Zn promoted electron transport chain (ETC) activities and ATP biosynthesis via increased expression levels of MDHs and ATPases.

In conclusion, Zn supplementation increased Cd accumulation in aboveground parts and reduced the production of O_2_^−^ and H_2_O_2_ in the leaves of *S. nigrum* under Cd treatment. Our comparative analysis of transcriptomes and proteomes provided molecular evidence for mechanisms of Cd transport and accumulation in shoots of *S. nigrum* exposed to Cd and Zn compound treatments. As hypothesized in Figure 8, Cd or Zn enters into a cell via transporters on the plasma membrane, such as ZIPs, Nramp1, and YSL2, and then small molecular proteins or peptides, such MTs and GSTs, are induced by Zn or Cd for antioxidant protection or chelation with excess metal ions. The Cd-GSH complexes are then transported to vacuoles for sequestration, or to cell walls for xylem transport by ABCs and OPTs. In support of that scenario, the DEGs of *ZIPs*, *Nramp1*, *YSL2*, *GSTs*, *ABCs*, and *OPTs* were more upregulated in Zn100+Cd100 than in Cd100. In addition, the Zn addition promoted ETC activities and ATP and chlorophyll biosynthesis by increasing DEGs or DEPs of MDHs, ATPases, and chlorophyll a/b binding protein to alleviate Cd toxicity in *S. nigrum*. The results provide a theoretical basis for the application of *S. nigrum* in the phytoremediation of soil polluted with Cd and Zn compounds.

## Data Availability

All datasets presented in this study are included in the article and Supplementary Materials. The raw datasets generated during the current study are available in ProteomeXchange Consortium (http://proteomecentral.proteomexchange.org, accessed on 9 February 2023) via the iProX partner repository with the project PXD039977.

## Supplementary Materials

The following supporting information can be downloaded at https://www.mdpi.com/article/10.3390/plants13172528/s1, Table S1: Transcriptome sequencing data of *S. nigrum* leaves under Zn and Cd treatment; Table S2: Primers of some genes in *S. nigrum* for qRT-PCR; Figure S1: Expression levels of DEGs involved in antioxidant protection in leaves of *S. nigrum* by transcriptome (a) and qRT-PCR (b); Figure S2: Expression levels of genes involved in cysteine (a) and glutathione (b) metabolism in leaves of *S. nigrum* by transcriptome.
