# Supplementary material for: Zinc Enhances Cadmium Accumulation in Shoots of Hyperaccumulator Solanum nigrum by Improving ATP-Dependent Transport and Alleviating Toxicity
Source: Plants (Basel). 2024 Sep 9;13(17):2528. doi: 10.3390/plants13172528 (PMC11397465; doi:10.3390/plants13172528)
Supplement: Supplementary file 1 [file plants-13-02528-s001.zip › plants-3133908-supplementary.pdf]

Supporting Materials:

Zinc Enhances Cadmium Accumulation in Shoots of Hyperaccumulator *Solanum nigrum* by Improving ATP-dependent Transport and Alleviating Toxicity

Table S1. Transcriptome sequencing data of *S. nigrum* leaves under Zn and Cd treatment.

Samples	Read number	Base number	GC Content	%≥Q30
CK-1	32001719	9600515700	42.23	95.11
CK-2	29860166	8958049800	42.34	92.70
CK-3	26150731	7845219300	42.30	92.93
Zn-1	20303904	6091171200	42.40	95.34
Zn-2	22102926	6630877800	44.09	95.43
Zn-3	21541203	6462360900	42.50	95.08
Cd-1	26268369	7880510700	42.52	95.46
Cd-2	20641928	6192578400	42.86	95.22
Cd-3	23629249	7088774700	42.43	95.13
ZnCd-1	28995486	8698645800	42.55	91.71
ZnCd-2	22990500	6897150000	42.73	92.44
ZnCd-3	23204534	6961360200	42.63	91.49

Table S2. Primers of some genes in *S. nigrum* for qRT-PCR.

gene names	Forward (from 5′ to 3′)	Reverse (from 5′ to 3′)
MT2a	GGACATTGAGAAGTCCACTACC	CACCTGCTGCTTTCTCATCTA
MT2b	GTCTTGCTGTGGAGGAAACT	TCAGTGATTGTGCTGCTCTC
MT2bX1	TCCACTACCCTCACCATCAT	CCCTCCTTCTGTTGCTTTCT
MT2c	TCCACTACCCTCACCATCAT	CTTGCATCCATTCCCTCCTT
ZIP2	AAGTCGAAGTGGATGAAGAAGG	GAGTGGAAACACAATGCAAGAA
ZIP10	GTTCTATCCCGGCCCTAAAC	TCCGGTAACACGTGCATAAA
COP5.1	AACTCGGCGATTGGGTATTT	CGCCGATCCTGAAGAACAA
COP6.3	GTCACCACCATCACCACAAA	TGGCCAGCCTGAGAATAGA
YSL2	CGGTGTTGTAGCAGGACTTAT	GGTCAGATAGCTCGTCTTGAAA
Sultr1	AAGGTCCCTGGCATACTTATTG	GTCTTCGTCAGTTAGCCATCTT
OPT3.1	CTTCTTCTCGTGGGTCTGTTG	CCAGCCCAATCAAGGGTAAA
PTR1	GTTTGTGTGCTGCCCTATCT	CCCATTCCTCGTGCTAATATCC
NRT2.5	CCGCCTCTTCTCCCAATTATC	TCACAAGCTGTTCCCATAGC
NRT2.7	GTGTTGTTGGGCTTGCTAATG	CCACGAAATGAACGAAGGAATG
ABCC15	GTACAATACAGCCCTGATCTCC	GACTTCCCACTACCTGTTCTTC
EF1α	CAAGATCCCATTCGTCCCCAT	GGTCAAGAGCCTCAAGGAGAGTT

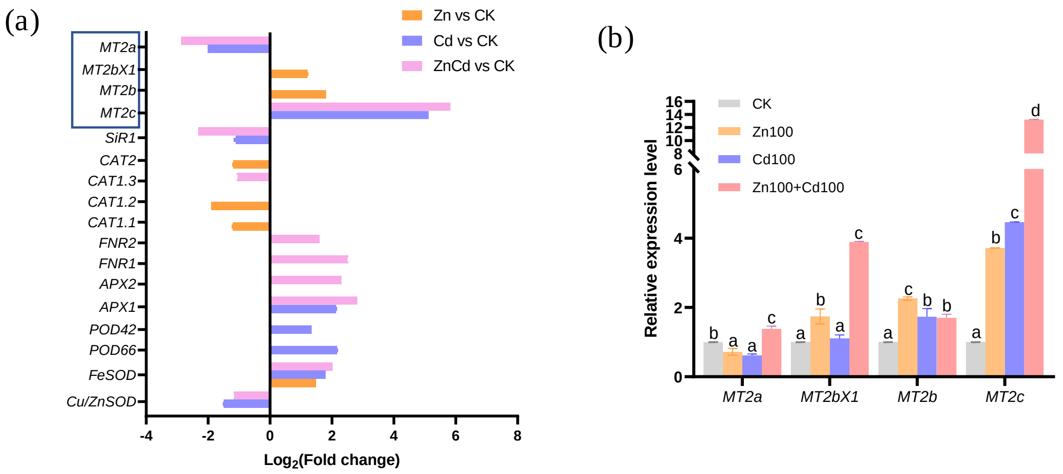

Figure S1. Expression levels of DEGs involved in antioxidant protection in leaves of *S. nigrum* by transcriptome (a) and qRT-PCR (b). Plant was exposed to a complete Hoagland solution (CK) or with 100 $\mu mol\cdot L^{-1}$ Zn (Zn100), 100 $\mu mol\cdot L^{-1}$ Cd (Cd100) and 100 $\mu mol\cdot L^{-1}$ Zn +100 $\mu mol\cdot L^{-1}$ Cd (Zn100+Cd100) for 10 days. Expression level of genes by transcriptome was showed use Log_2(Fold change) between sample sets (Zn vs CK, Cd vs CK and ZnCd vs CK). Relative expression level of MT genes by qRT-PCR denoted by different letters refer to the significant differences ($p < 0.05$, Duncan's test). *MT2a*, *MT2b*, *MT2bX1*, *MT2c*: metallothionein family members; *SiR*: sulfite reductase; *CAT*: catalase; *FNR*: ferredoxin-NADP reductase; *APX*: L-ascorbate peroxidase; *POD*: peroxidase; *FeSOD*: iron superoxidase dismutase; *Cu/ZnSOD*: copper, zinc superoxidase dismutase.

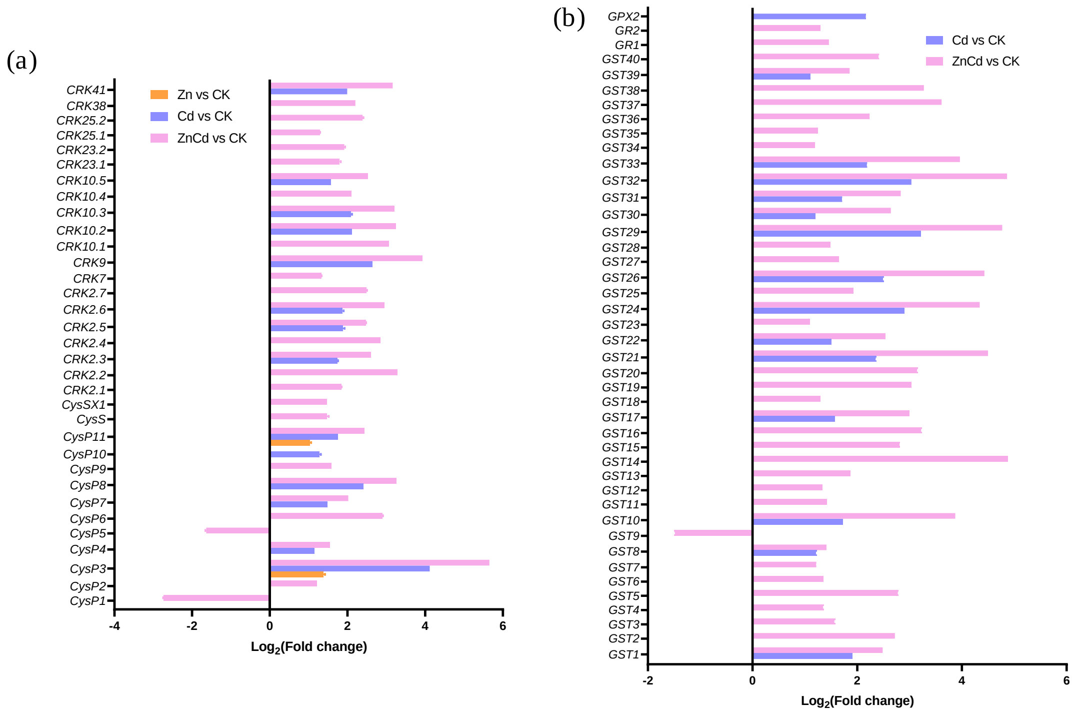

Figure S2. Expression levels of genes involved in cysteine (**a**) and glutathione (**b**) metabolism in leaves of *S. nigrum* by transcriptome. Plant was exposed to a complete Hoagland solution (CK) or with 100 $\mu mol \cdot L^{-1}$ Zn (Zn100), 100 $\mu mol \cdot L^{-1}$ Cd (Cd100) and 100 $\mu mol \cdot L^{-1}$ Zn+100 $\mu mol \cdot L^{-1}$ Cd (Zn100+Cd100) for 10 days. Expression level of gene was showed use Log_2(Fold change) between sample sets (Zn vs CK, Cd vs CK and ZnCd vs CK). *GRK*: cysteine-rich receptor-like protein kinase; *CysS*: cysteine synthase; *CysP*: cysteine proteinase precursor; *GPX*: glutathione peroxidase; *GR*: glutathione reductase; *GST*: glutathione S-transferase.
